# Recurrent Acute Pancreatitis Revealing Pancreatic Infiltration by CD20-Positive B-cell Lymphoma: A Diagnostic Pitfall in Extranodal Lymphoma

**DOI:** 10.7759/cureus.105934

**Published:** 2026-03-26

**Authors:** Daniela Prado Escobar, Claudia Pedreira, Kian Memari, Shane Williams, Lissette P Lazo, Peter Cohen

**Affiliations:** 1 Internal Medicine, Nova Southeastern University Dr. Kiran C. Patel College of Osteopathic Medicine, Clearwater, USA; 2 Family Medicine, Nova Southeastern University Dr. Kiran C. Patel College of Osteopathic Medicine, Davie, USA; 3 Family Medicine, Palmetto General Hospital, Hialeah, USA

**Keywords:** b-cell lymphoma, extranodal lymphoma, pancreatic infiltration, r-chop chemotherapy, recurrent pancreatitis, retroperitoneal lymphadenopathy

## Abstract

Acute pancreatitis most commonly results from gallstone disease, alcohol use, metabolic abnormalities, or medication effects. Malignant infiltration of the pancreas is a rare cause of pancreatic inflammation and may present with symptoms indistinguishable from more typical forms of pancreatitis. Pancreatic involvement by lymphoma is particularly uncommon and may mimic pancreatic neoplasms or recurrent inflammatory pancreatic disease. We present the case of a 29-year-old woman with a recently diagnosed CD-20-positive B-cell lymphoma who developed recurrent severe abdominal pain and markedly elevated pancreatic enzymes shortly after hospitalization for acute pancreatitis. Imaging revealed bulky retroperitoneal lymphadenopathy, bilateral renal enlargement, biliary ductal dilatation, and findings suspicious for pancreatic head involvement. Histopathologic evaluation of a retroperitoneal lymph node biopsy confirmed CD20-positive B-cell lymphoma. The patient underwent placement of a chemotherapy port and initiation of rituximab, cyclophosphamide, doxorubicin, vincristine, and prednisone (R-CHOP), with subsequent clinical improvement. This case highlights pancreatic infiltration by lymphoma as a rare cause of recurrent pancreatitis and underscores the importance of considering hematologic malignancy in patients presenting with atypical pancreatic inflammation or unexplained lymphadenopathy.

## Introduction

Acute pancreatitis is a common gastrointestinal emergency most often caused by gallstones, alcohol use, hypertriglyceridemia, medications, or metabolic derangements [1]. In uncommon cases, however, pancreatic inflammation may reflect malignant infiltration rather than a primary inflammatory pancreatic disorder [2].

Extranodal lymphoma refers to lymphomatous involvement arising outside the lymph nodes and occurs in approximately 30-40% of patients with non-Hodgkin lymphoma, with the gastrointestinal tract representing the most frequent site of extranodal disease [2]. Pancreatic lymphoma is rare and may occur either as primary pancreatic lymphoma, in which the dominant disease burden is centered in the pancreas, or as secondary pancreatic involvement, in which systemic lymphoma extends into pancreatic tissue or the pancreaticobiliary region [3,4]. Pancreatic involvement accounts for less than 2% of extranodal lymphomas and less than 1% of pancreatic malignancies [3,4].

Patients with pancreatic lymphoma may present with abdominal pain, obstructive jaundice, nausea, vomiting, weight loss, or, more rarely, acute pancreatitis [3-6]. For non-specialists, several imaging and clinical clues may raise suspicion for lymphoma over pancreatic adenocarcinoma, including bulky peripancreatic or retroperitoneal lymphadenopathy, renal or other extranodal involvement, relative preservation of peripancreatic vessels, and less prominent pancreatic duct obstruction than expected for the size of the lesion [5,7]. These distinctions are clinically important because pancreatic lymphoma is primarily treated with systemic therapy rather than pancreatic resection [3,4,8].

We present a case of recurrent acute pancreatitis that ultimately revealed pancreatic infiltration by CD20-positive B-cell lymphoma, illustrating how extranodal lymphoma may mimic more common pancreatic and biliary disease.

## Case presentation

A 29-year-old woman with a recent history of acute pancreatitis and acute kidney injury presented to the emergency department with sudden-onset severe abdominal pain and persistent nausea. The pain began earlier that day, was rated 10/10 in intensity, and radiated to the back. She reported multiple episodes of vomiting followed by persistent dry heaving.

Five days before presentation, she had been discharged after hospitalization for pancreatitis and renal dysfunction. During that admission, serum lipase had peaked near 8,000 U/L and subsequently decreased to approximately 200-800 U/L at discharge.

**Figure 1:**
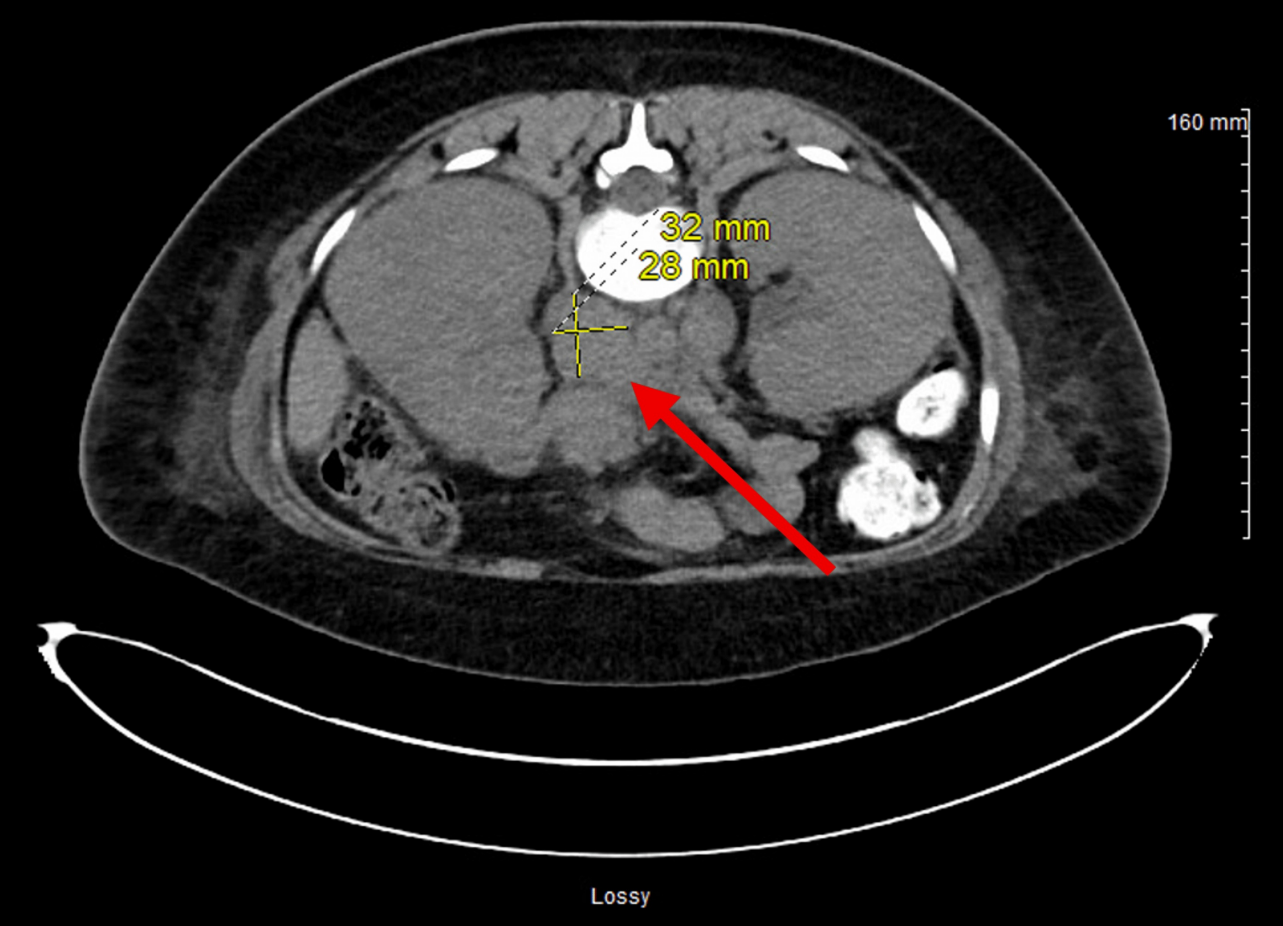
CT-guided biopsy of retroperitoneal lymphadenopathy CT-guided biopsy was performed in a 29-year-old female with abnormal bilateral renal appearance and interval development of retroperitoneal lymphadenopathy. Pre-procedure imaging demonstrated a 3.2 × 2.8 cm conglomerate of lymph nodes in the right retroperitoneum. The image shows successful needle placement targeting the retroperitoneal lymph node conglomerate for tissue sampling (red arrow).

During the prior hospitalization, imaging demonstrated retroperitoneal lymphadenopathy and bilateral renal enlargement (Figure 1). Biopsy of retroperitoneal lymph nodes revealed atypical lymphoid infiltration compatible with CD20-positive B-cell lymphoma. More specific subclassification beyond CD20-positive B-cell lymphoma was not available in the medical record.

On the current admission, laboratory evaluation demonstrated elevated lipase (2,203 U/L), severe transaminitis, normocytic anemia, and elevated creatinine (Table 1).

**Table 1:** Pertinent laboratory findings ALT, alanine transaminase; AST, aspartate aminotransferase

Laboratory test	Result	Units	Range	Clinical note/interpretation
Lipase	2,203	U/L	23-300	Markedly elevated, consistent with recurrent pancreatitis
Hemoglobin	11.8	g/dL	12.0-16.0	Mild normocytic anemia
Creatinine	2.1	mg/dL	0.52-1.04	Acute kidney injury/renal involvement concern
AST	2,621	U/L	10-40	Severe hepatocellular injury pattern
ALT	974	U/L	9-46	Severe hepatocellular injury pattern
D-dimer	13.49	ug/mL FEU	<0.50	Markedly elevated, nonspecific inflammatory/thrombotic marker

**Figure 2:**
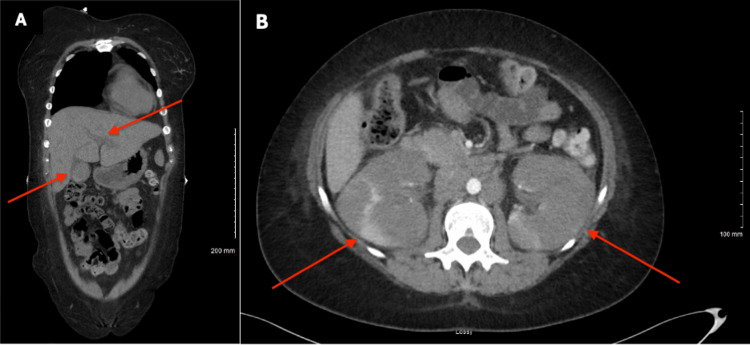
CT abdomen and pelvis without contrast demonstrating hepatobiliary and renal abnormalities (A) Coronal view showing intrahepatic ductal dilatation and increased density within the gallbladder lumen, suggestive of biliary sludge or gallbladder debris (red arrows). (B) Axial view demonstrating bilateral renal enlargement (red arrows). Imaging also revealed increased retroperitoneal soft tissue fullness compared with prior studies, consistent with progression of a known lymphoproliferative process.

**Figure 3:**
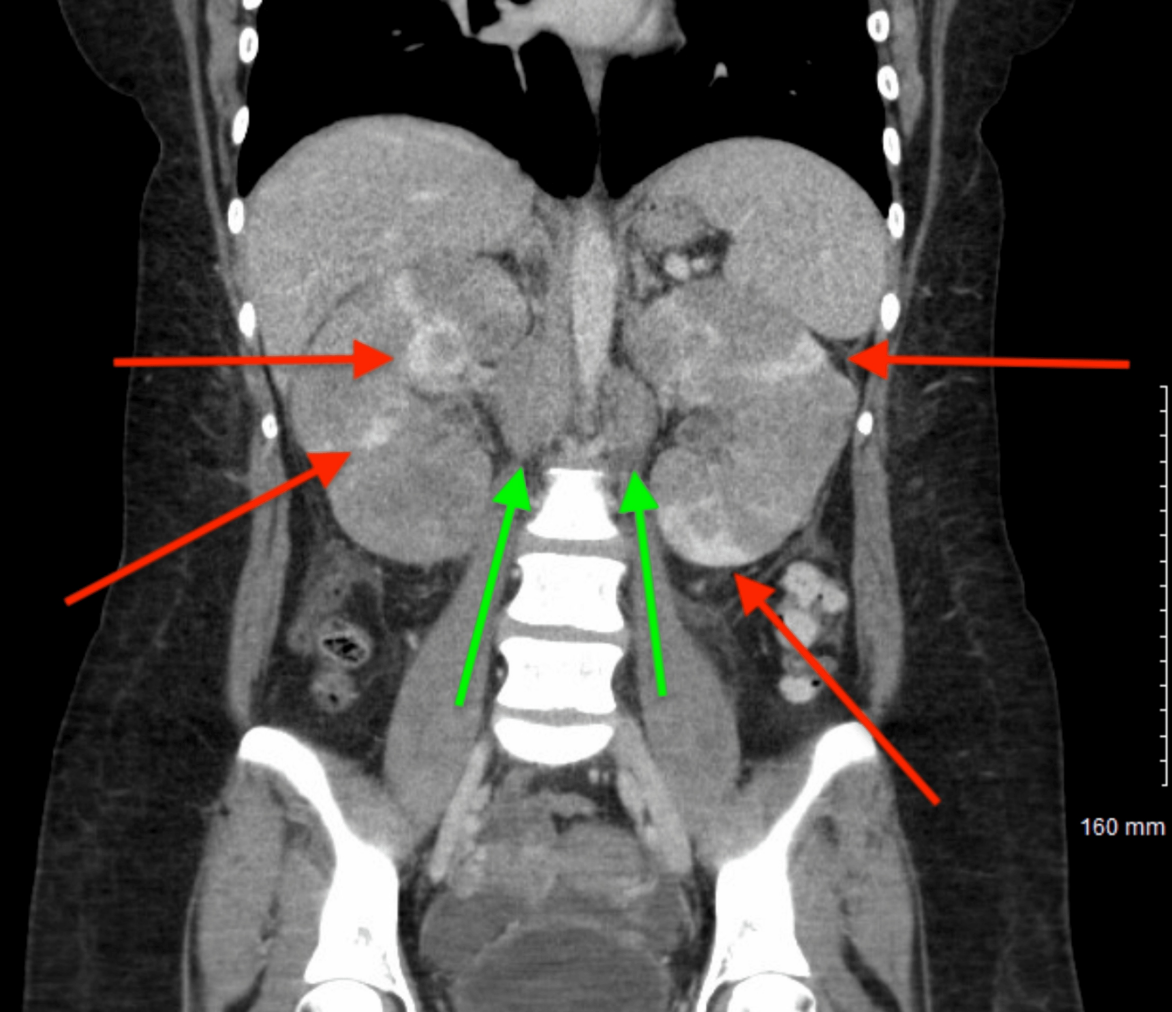
Contrast-enhanced CT abdomen and pelvis demonstrating renal cortical lesions and retroperitoneal lymphadenopathy Contrast-enhanced CT showing multifocal bilateral renal cortical hypoenhancing lesions with similar distribution compared with prior imaging (red arrows). There is also interval progression in the size and conspicuity of retroperitoneal lymph nodes, most prominent in the periaortic and aortocaval regions (green arrows), concerning for progression of an underlying lymphoproliferative process.

**Figure 4:**
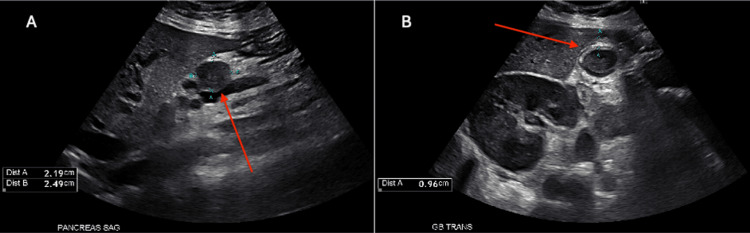
Abdominal ultrasound demonstrating pancreatic head lesion and biliary abnormalities (A) Sagittal view of the pancreas showing a complex echogenic lesion in the pancreatic head measuring approximately 2.2 × 2.5 × 2.3 cm (red arrow). The surrounding liver parenchyma demonstrates diffusely increased echogenicity, consistent with fatty infiltration. (B) Transverse view of the gallbladder demonstrating a dilated common bile duct measuring approximately 0.96 cm with associated gallbladder wall thickening and biliary sludge (red arrow), raising concern for biliary obstruction in the setting of pancreatic head involvement.

**Figure 5:**
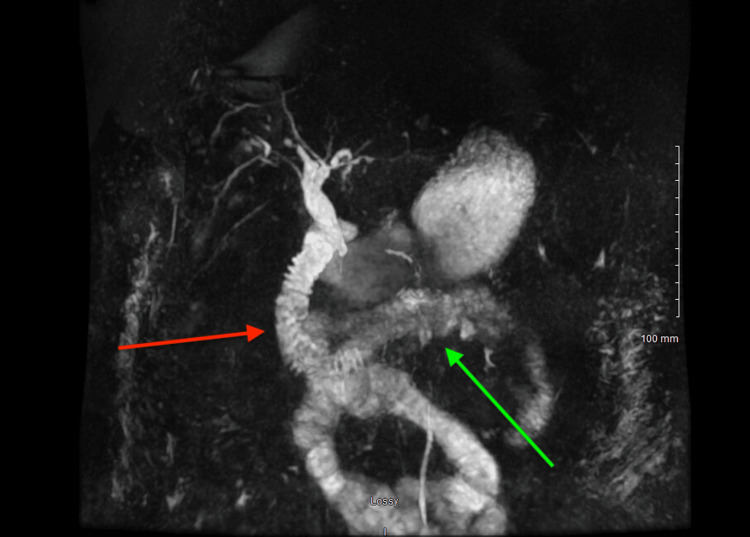
Magnetic resonance cholangiopancreatography demonstrating dilated common bile duct and pancreatic duct Magnetic resonance cholangiopancreatography demonstrating dilatation of the common bile duct (red arrow) and pancreatic duct (green arrow), findings suspicious for obstruction related to pancreatic head involvement.

CT of the abdomen demonstrated extensive retroperitoneal lymphadenopathy and bilateral renal enlargement suggestive of lymphoproliferative disease (Figure 2, Figure 3). Right upper quadrant ultrasound demonstrated gallbladder wall thickening with pericholecystic fluid, biliary sludge, and dilation of the common bile duct without visible gallstones (Figure 4). Magnetic resonance cholangiopancreatography demonstrated dilatation of both the common bile duct and pancreatic duct with findings suspicious for pancreatic head involvement (Figure 5).

Detailed physical examination findings, such as fever, documented weight loss, and palpable peripheral lymphadenopathy, were not fully recorded in the available chart. Clinically, however, the patient had recurrent severe epigastric pain radiating to the back with vomiting and objective biochemical evidence of pancreatitis.

After multidisciplinary consultation, a chemotherapy port was placed following cardiology clearance, confirming preserved ejection fraction. The patient received cycle 1 of rituximab, cyclophosphamide, doxorubicin, vincristine, and prednisone, along with tumor lysis prophylaxis using allopurinol and intravenous fluids. Her symptoms improved with treatment, and she was discharged with outpatient oncology follow-up.

## Discussion

This case illustrates an uncommon presentation of extranodal CD20-positive B-cell lymphoma manifesting as recurrent acute pancreatitis with suspected pancreatic infiltration. Although pancreatic lymphoma is traditionally discussed under the framework of primary pancreatic lymphoma, the imaging pattern in this patient, including bulky retroperitoneal lymphadenopathy and bilateral renal involvement, is more consistent with secondary pancreatic involvement by systemic lymphoma rather than a truly primary pancreatic lymphoma centered in the pancreas [3,4,9].

Pancreatic lymphoma is rare, whether primary or secondary. When it occurs, patients may present with abdominal pain, jaundice, nausea, vomiting, weight loss, or, less commonly, acute pancreatitis [3-6]. Several reported cases describe lymphoma-associated pancreatitis as the initial manifestation of either primary pancreatic lymphoma or secondary pancreatic infiltration, underscoring how easily this entity can be mistaken for gallstone pancreatitis or pancreatic adenocarcinoma [6,9]. Compared with typical pancreatic adenocarcinoma, lymphoma is more likely to present with bulky peripancreatic or retroperitoneal lymphadenopathy and less vascular encasement, and it may produce less ductal distortion than expected for the degree of soft tissue involvement [5,7].

Several mechanisms may explain pancreatitis in this setting. Pancreatic inflammation may result from direct infiltration of pancreatic tissue by lymphoma, compression of the pancreaticobiliary system by adjacent nodal disease, or combined mass effect at the pancreatic head [5,6,9]. In this case, MRCP and ultrasound suggested pancreatic head involvement with associated biliary and pancreatic ductal dilatation, supporting an obstructive or infiltrative mechanism. At the same time, biliary sludge and gallbladder wall thickening were also present, so a biliary contribution cannot be completely excluded. The clinical picture, therefore, strongly suggests lymphoma-related pancreatic involvement, but direct causality cannot be proven with absolute certainty in the absence of pancreatic tissue sampling.

A pancreatic biopsy was not performed because diagnostic tissue had already been obtained safely from the retroperitoneal lymph node conglomerate, confirming CD20-positive B-cell lymphoma, and because the combination of systemic disease burden, pancreatic head imaging abnormalities, and rapid need for treatment made an additional pancreatic procedure unlikely to change initial management. In this context, retroperitoneal node biopsy provided a lower-risk route to histologic diagnosis than pancreatic sampling. Endoscopic ultrasound-guided fine-needle aspiration or core biopsy remains an important option when pancreatic tissue diagnosis is needed or when the diagnosis remains uncertain after less invasive tissue acquisition [8].

This case also highlights the practical limits of the available staging and pathologic data. Baseline lactate dehydrogenase, PET-CT findings, and additional lymphoma markers such as Ki-67, CD10, BCL2, BCL6, or MYC were not available in the chart, and more specific subclassification beyond CD20-positive B-cell lymphoma was not reported. These missing data limit a more precise assessment of systemic tumor burden, biologic aggressiveness, and lymphoma subtype. Nevertheless, the clinical course, cross-sectional imaging, nodal biopsy, and subsequent response to rituximab-based chemotherapy strongly support lymphomatous pancreatic involvement as the dominant unifying diagnosis.

Treatment of pancreatic involvement by B-cell lymphoma is generally systemic rather than surgical. Rituximab-based combination chemotherapy, such as rituximab, cyclophosphamide, doxorubicin, vincristine, and prednisone, remains a standard first-line approach for many aggressive CD20-positive B-cell lymphomas and can lead to meaningful clinical improvement when diagnosis is established promptly [10]. In previously reported cases of lymphoma-associated pancreatitis, chemotherapy has likewise been associated with rapid symptomatic and radiographic improvement, in contrast to pancreatic adenocarcinoma, where surgical and oncologic pathways differ substantially [6,9].

The major diagnostic lesson of this case is that recurrent pancreatitis in a young patient without a classic risk profile should prompt broader investigation when imaging reveals extensive extrapancreatic lymphadenopathy, renal enlargement, or other lymphoproliferative features. Earlier recognition of lymphoma as a potential driver of pancreatic inflammation can expedite tissue diagnosis, avoid unnecessary pancreatic surgery, and facilitate timely systemic therapy. At the same time, clinicians should acknowledge alternative contributors such as biliary sludge when present and interpret imaging and pathology findings in an integrated, multidisciplinary fashion.

## Conclusions

Pancreatic infiltration by lymphoma is a rare but clinically important cause of recurrent pancreatitis. In patients with newly diagnosed lymphoproliferative disorders, unexplained abdominal pain and elevated pancreatic enzymes should prompt evaluation for extranodal pancreatic involvement.

In this case, pancreatic involvement was inferred from imaging findings, systemic disease distribution, and treatment response rather than direct pancreatic tissue confirmation. Although biliary sludge may have contributed to the clinical presentation, the overall pattern strongly supported lymphoma-related pancreatic disease. Early recognition of this possibility can reduce diagnostic delay and facilitate the timely initiation of systemic chemotherapy.
